# What is Criminal Rehabilitation?

**DOI:** 10.1007/s11572-020-09547-4

**Published:** 2020-10-03

**Authors:** Lisa Forsberg, Thomas Douglas

**Affiliations:** 1 British Academy Postdoctoral Fellow, Faculty of Law, University of Oxford, St Cross Building, St Cross Road, Oxford, OX1 3UL UK; 2 Oxford Uehiro Centre for Practical Ethics, Faculty of Philosophy, University of Oxford, Suite 8, Littlegate House, 16/17 St. Ebbe’s St., Oxford, OX1 1PT UK; 3 Somerville College, University of Oxford, Woodstock Road, Oxford, OX2 6HD UK; 4 Hugh Price Fellow, Jesus College, Turl Street, Oxford, OX1 3DW UK

**Keywords:** Criminal rehabilitation, Moral education, Moral improvement, Criminal justice, Reform

## Abstract

It is often said that the institutions of criminal justice ought or—perhaps more often—ought not to *rehabilitate* criminal offenders. But the term ‘criminal rehabilitation’ is often used without being explicitly defined, and in ways that are consistent with widely divergent conceptions. In this paper, we present a taxonomy that distinguishes, and explains the relationships between, different conceptions of criminal rehabilitation. Our taxonomy distinguishes conceptions of criminal rehabilitation on the basis of (i) the aims or ends of the putatively rehabilitative measure, and (ii) the means that may be used to achieve the intended end. We also explore some of the implications of each conception, some of the payoffs of a taxonomy of the kind we offer, and some areas for future work.

It is often said that the institutions of criminal justice ought or—perhaps more often—ought not to *rehabilitate* criminal offenders. Such claims can be found in academic literature—for example, from criminology and penal theory.^1^ They can also be found in policy documents and legal judgments.^2^ But what, exactly, does criminal rehabilitation consist in? The term is often used without a clear referent, and in ways that are consistent with widely divergent conceptions. As Ted Honderich notes, ‘a number of views [recommend] punishment or some other practice for dealing with crime on the ground that it will reform, correct, rehabilitate, treat, improve or cure offenders’, but ‘[o]ften these doctrines have been ill-defined’.^3^

This imprecision cannot be excused on the basis that, in practice, the boundaries of the concept of rehabilitation are intuitively clear, for there are, in fact, many grey zones. When prison authorities provide psychological therapies to prisoners suffering from depression, are they rehabilitating those prisoners? When a parole board requires that a paroled sex offender undergoes ‘chemical castration’, is it imposing a form of rehabilitation? Is imprisonment itself rehabilitative? The answers to these questions are, we think, not obvious.

In this paper, we present a taxonomy that distinguishes and explains the relationships between different conceptions of criminal rehabilitation.^4^ We also explore some of the implications of each conception, and some of the payoffs of a taxonomy of the kind we offer. The taxonomy distinguishes conceptions of criminal rehabilitation on the basis of (i) the aims or ends of the putatively rehabilitative measure, and (ii) the means that may be used to achieve the intended end. This two-dimension approach reflects the fact that, on some conceptions, rehabilitation is to be distinguished from other functions of criminal justice by the ends at which it aims, on others, it is to be distinguished by the means used to achieve this end, while on others still it is to be distinguished by the combination of means and ends that it deploys. Our main motivation for offering this taxonomy is the hope that explicitly separating distinct conceptions of criminal rehabilitation will serve as a first step towards remedying the unclarity that characterises much of the existing literature on rehabilitation. We hope, for example, that our taxonomy might help to clarify the scope of influential criticisms of criminal rehabilitation—it may allow us to precisely specify which practices are unjustified if these criticisms succeed.

Section one presents some of the reasons that a taxonomy of criminal rehabilitation (henceforth just ‘rehabilitation’) is needed. Section two illustrates some of the different ways in which rehabilitation is understood in the literature. Section three outlines five different conceptions of rehabilitation, distinguished from one another by the ends that they take rehabilitation to serve. Section four introduces means-based subvariants of the different conceptions identified in the preceding section. Section five explores some of the payoffs of our taxonomy of rehabilitation. Finally, section six identifies some areas for future work.

We remain neutral throughout on the role that rehabilitation should play in actual or ideal criminal justice systems. Though we are sympathetic to the view that criminal justice systems ought to rehabilitate, and this partly motivates our interest in the topic, we are not committed to this view, let alone to the stronger view that rehabilitation ought to be the *sole* or *primary* official function of criminal justice. We also take no view on whether, if criminal justice systems ought to rehabilitate, this rehabilitation ought to be conceived as an aspect of punishment, or as something that is done in place of or in addition to punishment. In addition, we leave it open whether traditional forms of punishment, such as incarceration, themselves qualify as instances or components of rehabilitation.

We will, from the outset, exclude from the category of rehabilitation all interventions that aim to prevent an individual from re-offending (i) by making it physically impossible for the person to re-offend (e.g. by physically separating the offender from potential victims, or killing the offender), or (ii) purely by introducing disincentives or incentives. This is because we wish to maintain a distinction between rehabilitation and two forms of intervention with which it is often contrasted: incapacitation and deterrent punishment. However, in the interests of offering an inclusive taxonomy, we will otherwise start from a broad working conception of rehabilitation that includes all interventions that have commonly been referred to as ‘rehabilitation’, as well as some that we think are sufficiently similar to those practices that they might, without obvious error, be picked out using that label.

## Why Conceptual Clarity is Needed

We need a taxonomy of criminal rehabilitation in order to protect against the conflation and confusion of different conceptions of rehabilitation. Why do we need this? There are at least five reasons.

First, the thought that criminal offenders ought to be rehabilitated has exerted a strong influence on the design of many criminal justice systems, including some not generally thought of as rehabilitation-focused, such as the United States’ system. This can be seen, for example, in the language used to describe parts of the criminal justice system: US prisons are, for instance, often referred to as ‘correctional facilities’, and their staff as ‘correctional officers’.^5^ Having a clear view of the conception(s) of rehabilitation that informed their development could help us better understand the historical development of such criminal justice systems.

Second, although pure rehabilitation theories according to which rehabilitation is the sole legitimate function of criminal justice are no longer popular in moral and legal philosophy, the rehabilitation of offenders—or something akin to it—does, as we will discuss further below, play some role in many currently influential theories, such as those defended by Robert Nozick, Antony Duff, and Victor Tadros.^6^

Third, notwithstanding the turn against purely rehabilitative theories of criminal justice, our criminal justice systems do, as a matter of fact, continue to prominently pursue what could be aptly described as rehabilitation.^7^ Whether or not we think that our criminal justice system ought to be in the business of rehabilitation, they *are* in this business, and criminal justice practitioners generally acknowledge this. Rehabilitation programmes, broadly construed, are in place in prisons in most jurisdictions in Europe and North America. The nature and purpose of such programmes vary according to type of offence and the offender's perceived needs, but include education, vocational training, psychological/behavioural interventions, and interventions addressing offenders’ addiction problems. The United Kingdom currently operates rehabilitation programmes designed to reduce offenders’ aggressive behaviour,^8^ treat alcohol and substance abuse related to offending behaviour,^9^ and target some particular types of offending such as domestic abuse and sexual offences.^10^ The means used to achieve these ends are generally counselling-based, but can also include pharmaceutical interventions (especially when targeting addiction-related offending and sex offending, in relation to which methadone maintenance therapy and anti-libidinal interventions are sometimes employed). The European Court of Human Rights has stated that signatory member states have a positive obligation to foster the rehabilitation of criminal offenders, and that criminal justice systems should be designed with this aim in mind.^11^ Given that we apparently *are* attempting to rehabilitate criminal offenders, we should get clear on what exactly rehabilitation comprises.

Fourth, a better understanding of rehabilitation may allow us to better appraise moral objections to rehabilitation and to rehabilitative theories of criminal justice. Rehabilitation fell out of favour in moral and legal philosophy due in part to moral concerns, for example, regarding its putative failure to treat offenders as moral agents responsible for their conduct (the ‘theoretical objection’).^12^ However, rehabilitation has received insufficient attention from philosophers and arguments for it are often not presented charitably.^13^ We suspect that a failure to clearly describe rehabilitation may have led to its being prematurely dismissed by some.

Finally, a better understanding of rehabilitation may help us determine the extent to which rehabilitative theories are capable of overcoming the other main set of concerns that caused them to fall out of favour: empirical worries to the effect that measures taken aimed at rehabilitating offenders were of limited effectiveness (the ‘empirical objection’).^14^ The ineffectiveness of rehabilitation has been questioned,^15^ and even if currently available modes of rehabilitation—such as counselling—are indeed ineffective, it is possible that future modes—which might combine traditional interventions with interventions acting directly on offenders’ brains—will be more effective.^16^ To assess the empirical objection, both in relation to current and potential future interventions, we need a yardstick against which effectiveness can be measured—that is, we need to know what rehabilitation is and what it aims to achieve.

## Divergent Conceptions of Criminal Rehabilitation in the Literature

Though rehabilitation has been an influential concept in debates on criminal justice, it is often not properly defined or elucidated.

Some authors are careful to distinguish between ‘reform’ and ‘rehabilitation’. As some characterise this distinction, reform seeks to alter character traits, motivations or dispositions, whereas rehabilitation aims at ‘improvement of … skills, capacities, and opportunities’.^17^ Others understand reform as the historically prior practice of providing ‘opportunities for education and contemplation in support of the reform of one’s moral character’ and rehabilitation as the more recent (twentieth century) practice of using (primarily psychological) interventions aimed at ‘correcting offenders personality traits, behaviours or attitudes’.^18^ But not all employ this distinction or indeed agree that such a distinction can or should be made. We will use rehabilitation to refer to both what has been called rehabilitation and what has been called reform.

Steven Sverdlik notes that ‘[t]he history of reformist thinking about state punishment is confusing, in part because of terminological issues’.^19^ Plato and Hegel have been taken to be early proponents of reform or rehabilitation theories, but neither Plato nor Hegel uses a term that would be translated as ‘reform’ or ‘rehabilitation’ to refer to his view.^20^ Jeremy Bentham and A. C. Ewing both use the term ‘reform’, which they argue is at least part of what criminal justice should aim to achieve,^21^ but more contemporary defenders of what some would regard as varieties of rehabilitation or reform often explicitly reject these labels. Herbert Morris, Jean Hampton and Duff have all been characterised as defenders of reform or rehabilitation,^22^ but all reject one or both of these labels being applied to their theories. Morris rejects both the ‘reform’ and ‘rehabilitation’ labels,^23^ and Hampton takes care to distinguish her theory from ‘rehabilitative’ alternatives.^24^ Duff refers to the ‘reform’ of offenders as an aim of criminal punishment, but sees punishment as encouraging *self*-reform, which he appears to understand as distinct from reform *simpliciter*, as understood in traditional rehabilitation theories. He rejects the unqualified ‘reform’ and ‘rehabilitation’ labels since he takes these to be compatible with or to include interventions that make offenders law-abiding in ways that bypass or undermine their moral agency. His theory requires active engagement of the offender’s moral agency.^25^

More recent literature concerned with the moral permissibility of using so-called neurointerventions, such as brain-active drugs, in crime-prevention employs various conceptions of rehabilitation and often expresses ambivalence about how it should be understood, and/or a reluctance to commit to any univocal conception. For example, Lene Bomann-Larsen refers to ‘voluntary rehabilitation programs aiming at correcting undesirable behaviour’ or ‘to change [offenders’] undesirable behavioural pattern’.^26^ Elizabeth Shaw refers to interventions to ‘develop more effective ways of re-integrating offenders back into society’ and avoid ‘reconviction’.^27^ One of the present authors (Douglas) employs a disjunctive definition on which rehabilitation aims either at ‘making offenders less disposed to offend’, or at ‘moral improvement’.^28^ In this more recent literature, there is an on-going debate regarding whether interventions intended to rehabilitate offenders need to engage the offender’s rational capacities in order to be morally permissible. This discussion mirrors some of the earlier literature on whether criminal rehabilitation ought to be pursued through reason-engaging means.^29^

It is not just in the philosophical literature that criminal rehabilitation is often not clearly defined, or is used in ways that are consistent with divergent conceptions. Fergus McNeill notes that also in the criminological literature, ‘[b]oth as a set of concepts and as a set of practices, rehabilitation is a “tangle”’.^30^ Peter Raynor and Gwen Robinson suggest that, ‘despite the longevity and continuing relevance of the concept of rehabilitation in the context of offending, it has rarely been “unpacked” or examined critically’ and that ‘it is quite common to come across “offender rehabilitation” in both academic and policy contexts with no accompanying definition of the term’.^31^ Sonja Meijer argues that ‘rehabilitation remains vague’ and that ‘[i]nterpretations … differ between the disciplines and professional groups … (law, criminology and social work), but also within these groups’ and across jurisdictions.^32^

In what follows, we develop a taxonomy that clarifies how different conceptions of rehabilitation (broadly understood) differ and overlap with regard to the posited aims of rehabilitation, and the means via which they allow these aims to be pursued.

## Five Conceptions of Criminal Rehabilitation

We will start by distinguishing five conceptions of rehabilitation on the basis of their aims.

Consider first one rather ‘thin’, non-normative, conception of rehabilitation:

*Rehabilitation as anti*-*recidivism.* An intervention *I* administered by a criminal justice system to offender *O* in response to *O*’s offence is an instance of rehabilitation just in case (1) it is intended to reduce the likelihood that *O* will re-offend, (2) other than by reducing *O*’s capacity to reoffend, disincentivising re-offending by *O*, or incentivising non-offending by *O.*

The aim of reducing the likelihood of recidivism need not, we take it, be the ultimate goal of an intervention in order for it to qualify as rehabilitative on this conception. The ultimate goal may, for instance, be to protect third parties from harm, to promote public safety, to facilitate earlier release of the offender from prison, or simply to maximise aggregate utility. The aim of reducing the likelihood of recidivism also need not, we take it, be the immediate goal of the intervention. The intervention may, for instance, be intended to promote empathy, self-control, or introspection, with the reduced likelihood of offending being an intended effect of the realisation of that immediate aim.

Note that, on *rehabilitation as anti*-*recidivism,* rehabilitation may share with incapacitation and specific deterrence the aim of preventing people from committing future crimes, so its aim is not a distinctive feature. Rather, its distinctive feature lies in how it gets there, that is, in the means used to achieve this end. Incapacitation seeks to reduce the likelihood of recidivism through rendering it physically impossible, for example, by separating the offender from potential victims, or killing the offender. Special deterrence seeks to reduce the likelihood of re-offending by disincentivising it. Rehabilitation, by contrast, employs other means: most likely, the alteration of the offenders’ intrinsic dispositions.^33^

The anti-recidivist conception of rehabilitation is commonly endorsed, at least implicitly, in policy documents. For example, the Ministry of Justice in the UK uses recidivism as the outcome measure for assessing the effectiveness of interventions they refer to as rehabilitative.^34^

We might also consider a broader alternative to *rehabilitation as anti*-*recidivism*:

*Rehabilitation as harm*-*reduction*: An intervention *I* administered by a criminal justice system to offender *O* in response to *O*’s offence is an instance of rehabilitation just in case (1) it is intended to prevent harmful conduct *by O* (restricted to the kinds of harms that are legitimately the business of the criminal law), (2) other than by reducing *O*’s capacity to engage in such conduct, disincentivising such conduct by *O*, or to incentivising less harmful conduct by *O.*

This account requires some clarification.

First, for the purposes of *rehabilitation as harm*-*reduction*, we take harmful conduct to include conduct with negative effects on the wellbeing of others; on some subvariants of the view, it might also include harm to the offender himself.

Second, as our parenthetical rider indicates, the concept of harm, for the purposes of this account, will need to be restricted. Not all harms, even serious ones, are properly the target of the criminal law, and thus criminal rehabilitation. It is doubtful that we would classify an attempt to prevent an offender from cheating on his partner as rehabilitative. Moreover, even harms that are within the domain of criminal law may be too distant from the crime that has been committed to qualify as a proper target of an attempt at rehabilitation. It is, for instance, doubtful whether we would characterise an attempt to prevent a murderer from committing tax fraud as rehabilitative. Perhaps, to qualify as rehabilitation an intervention must target ‘harmful conduct’ relevantly similar to the offending behaviour of which the offender has been convicted.

Third, as with *rehabilitation as anti*-*recidivism*, we do not require that harm-reduction must be the immediate or ultimate goal of an intervention for it to qualify as rehabilitation on this view; it must simply be *a* goal.

*Rehabilitation as harm*-*reduction* seems to be deployed by Sverdlik in his defence of rehabilitative punishment. Sverdlik holds that punishment can be justified even when it does not have any general deterrent effects, because it may rehabilitate the offender—that is, reduce the likelihood that the offender will perform actions that ‘either cause serious setbacks to well-being, or pose a great risk of doing so’.^35^ Sverdlik sees rehabilitation as something that should aim at improving offenders’ responsiveness to prudential and moral reasons, however he appears to think of improving reasons-responsiveness as a means to the further end of diminishing social costs, rather than as an end in itself.^36^

An alternative to *rehabilitation as anti*-*recidivism* and *rehabilitation as harm*-*reduction* is:

*Rehabilitation as therapy.* An intervention *I* administered by a criminal justice system to offender *O* in response to *O*’s offence is an instance of rehabilitation just in case it is intended to cure or ameliorate a mental deficit in *O* that is understood by the intervener (1) to have causally contributed to *O*’s past offence(s), or (2) to predispose *O* to further offending.

‘Mental deficit’ can be understood in either of two different ways: as referring to a mental illness or disorder, or as referring to some defect in the capacities relevant for criminal responsibility, such as capacities for rational agency. The first might aptly be described as a ‘psychiatric’ understanding, since it equates the goals of rehabilitation with those of clinical psychiatry, whereas the second might be labelled a forensic understanding. There will likely be a large overlap in these two understandings, but we take it to be plausible that some mental disorders do not diminish rational capacities, and some diminishments in rational capacity to not constitute mental disorders.

On *rehabilitation as therapy*, and especially on the psychiatric understanding of it, the aims of rehabilitation overlap with those of clinical medicine (and more specifically, given the focus on mental illnesses and deficits, clinical psychiatry). As with standard medical treatments, the aim of curing or ameliorating the deficit may be instrumental to the further aim of benefitting the individual. However, other further aims are also possible. These may include, for example, preventing re-offending, protecting the public, or advancing the social good. If the further aims of the intervention include preventing recidivism or harmful conduct, then the intervention will qualify as rehabilitation on both *rehabilitation as therapy* and one or both of the accounts we offered above.

Bertrand Russell appears to have had something like *rehabilitation as therapy* in mind when he wrote that

When a man is suffering from an infectious disease, he is a danger to the community, and it is necessary to restrict his liberty of movement. But no one associates any idea of guilt with such a situation. On the contrary, he is an object of commiseration to his friends. Such steps as science recommends are taken to cure him of his disease, and he submits as a rule without reluctance to the curtailment of liberty involved meanwhile. The same method in spirit ought to be shown in the treatment of what is called ‘crime’.^37^

We think that *rehabilitation as therapy* can also be attributed to some who take themselves to be critics of rehabilitation. For example, Jean Hampton distinguishes her moral education theory of punishment from rehabilitative views by noting that her theory ‘does not perceive punishment as a way of treating a “sick” person for a mental disease, but rather as a way of sending a moral message to a person who has acted immorally and who is to be held responsible for her actions’.^38^ This suggests that she endorses a therapeutic conception of rehabilitation and denies that her own favoured form of punishment is rehabilitative on the basis that it is non-therapeutic. Herbert Morris also seems to endorse *rehabilitation as therapy* in characterising his own view as non-rehabilitative. He states that ‘[i]t is not one’s health; it is not even one’s moral health with respect to any particular matter that is sought to be achieved; it is one’s general character as a morally autonomous individual attached to the good’.^39^

It is, however, tempting to think of Hampton and Morris not as opponents of rehabilitation, but as proponents of a particular, non-therapeutic, kind of rehabilitation,^40^ namely:

*Rehabilitation as moral improvement.* An intervention *I* administered by a criminal justice system to offender *O* in response to *O*’s offence is an instance of rehabilitation just in case it is intended to morally improve *O*.

This is a thicker conception of rehabilitation than the ones we have previously considered, which have all been ‘thin’, in the sense that they characterise the goals of rehabilitation in non-normative terms, or at least in terms that can plausibly be understood as non-normative.^41^

Hampton maintains that ‘punishment is justified as a way to prevent wrongdoing insofar as it can teach both wrongdoers and the public at large the moral reasons for choosing not to perform an offense’.^42^ As we have seen, she does not regard punishment of this sort as rehabilitative, suggesting that she would reject *rehabilitation as moral improvement* as an account of the nature of rehabilitation.^43^ However, those who characterise Hampton as a proponent of rehabilitation may do so because they, in contrast to Hampton, endorse *rehabilitation as moral improvement*, or something close to it. From here on, we will accept the position of those (including Sverdlik) who characterise Morris and Hampton’s views as rehabilitative.^44^

Others have endorsed *rehabilitation as moral improvement* too. For example, Duff appears to have something like this conception in mind when he uses the term ‘moral rehabilitation’ to describe the kinds of changes at which his preferred type of communicative punishment aims.^45^ Jeffrey Howard’s moral fortification view is an explicit defence of rehabilitation that endorses *rehabilitation as moral improvement*, or something close to it—he aims to ‘resuscitate the rehabilitative approach to criminal justice’^46^ by developing a conception of rehabilitation that is immune to the criticism that rehabilitation fails to respect offenders as moral agents responsible for their conduct. Howard argues that offenders have an obligation, owed to other moral agents, to rehabilitate themselves, where rehabilitation is understood to consist in enhancing the dependability of one’s moral capacities.^47^

Whether or not they take themselves to be defending a variant of rehabilitation, those who defend the moral improvement of offenders as a legitimate goal of criminal justice understand moral improvement in different ways, and we might recognise these differences by distinguishing a number of different variants of *rehabilitation as moral improvement.* These variants share a commitment to a specific kind of end, that is, making the offender morally better, but differ in their understanding of what becoming morally better consists in (the nature of moral improvement), and on what sorts of moral improvement rehabilitation may legitimately aim at (the scope of legitimate moral improvement). On the nature of moral improvement we can, for example, distinguish between views according to which moral improvement consists in the acquisition of more justified moral beliefs, more morally virtuous character traits, more praiseworthy moral motives, or more morally desirable actions.^48^ On the scope of legitimate moral improvement, we can distinguish between attempts to morally improve a person with respect to the particular type of conduct for which the individual has been convicted, or more globally.

It has been argued that Hampton and Morris have in common that ‘the psychological changes in offenders that they are interested in promoting are, roughly, these: becoming convinced that one’s action was wrong; feeling guilty for performing it; resolving not to do it again’, and Howard and Duff hold views that are similar with respect to the kinds of changes they believe should be promoted.^49^ There appears then to be much agreement on the nature of moral improvement, but there are also important differences between their accounts, in particular regarding the scope of legitimate moral improvement.^50^

Morris favours a view on which rehabilitative measures may permissibly aim at a global kind of moral improvement.^51^ On his view, we should provide an offender with a form of moral education that helps him develop into ‘an autonomous individual freely attached to that which is good’.^52^ The particular good aimed at is a moral good, which has several parts of which the main ones are: ‘that one feel contrite, that one feel the guilt that is appropriate to one’s wrongdoing, that one be repentant, that one be self-forgiving, and that one have reinforced one’s conception of oneself as a responsible being’.^53^

On Howard’s view, the scope of moral improvement sought may be somewhat more local: offenders ought (as a matter of what they owe to their fellow moral agents) to take measures to reduce the likelihood that they will commit further criminal wrongs by undertaking measures aimed at fortifying their moral capacities and in particular their sense of justice.^54^

On Hampton’s view, interventions should do more than merely deter ‘THE offender’ from committing certain offences; they should also provide him with moral reasons for *choosing* to refrain from committing such offences.^55^ In this way, moral education imparts on offenders moral knowledge that will help them choose to do what is right. By ‘certain offences’ and ‘such offenses’, we mean offenses of the kind for which the offender is now being punished. Hampton states, for example, that ‘our principal concern as we punish is to get the wrongdoer to stop doing *the immoral action* by communicating to her that her offense was immoral’.^56^ This suggests a narrower understanding of the legitimate scope of rehabilitation; rehabilitation should only or at least mainly target moral improvements relevant to the particular sort of criminal activity that has been committed.

Similarly, Duff defends what some see as a rehabilitation-based account of criminal justice aimed at moral improvement^57^ on which it is not permissible for moral improvement to take a focus that is too global or wide-ranging.^58^ Duff insists that criminal justice ‘can properly insist on addressing only those aspects of [an offender’s] conduct or attitudes that constituted her crime’.^59^

As with the aims invoked by thin conceptions of rehabilitation, the aim of moral improvement may, on *rehabilitation as moral improvement*, be proximal to some further aim, such as the promotion of offender wellbeing, the social good, the non-instrumental value of being morally good (or the non-instrumental value of becoming morally better), or some combination of these. It may also be distal to some more immediate aim, such as the promotion of offender empathy, self-control, self-understanding, or introspection. Proponents of rehabilitation, as conceived in *rehabilitation as moral improvement*, typically assume some non-instrumental value to moral improvement.

Our fifth and final conception of rehabilitation is:

*Rehabilitation as restoration.* An intervention *I* administered by a criminal justice system to offender *O* in response to *O*’s offence is an instance of rehabilitation just in case it is intended to restore *O*’s moral or social relationships or standing.

On this conception, rehabilitation is a matter of restoring the offender’s social or moral standing in society or his social or moral relations with others, or fostering the capacities needed for such restoration. This could include social and vocational capacities as well as moral ones.^60^ On one variant of this conception, rehabilitation aims at restoring the offender’s *moral* relationships or standing. On this variant, criminal rehabilitation is in some respects akin to the payment of compensatory damages at tort law; its concern is to bring it about that the offender compensates his victim, pays off a moral debt owed to his victim, corrects the wrong committed, or restores the moral balance between offender and victim. On another variant, rehabilitation aims to restore the offender’s* social* relationships or repair a social injury, by, for example, helping the offender (re)establish friendships, family bonds, and relationships with others (including victims). A third, hybrid variant would understand rehabilitation as aiming at the restoration of both moral and social relationships.^61^ This seems to be the most commonly held variant of the view.

Proponents of *rehabilitation as restoration* include Margaret Fry, who in her advocacy for penal reform emphasised the rehabilitative potential of offenders paying damages to their victims, arguing that ‘repayment is the best first step towards reformation that a dishonest person can take’.^62^ Lucia Zedner holds that ‘criminal justice should be less preoccupied with censuring the code-breakers and focus instead on the process of restoring individual damage and repairing ruptured social bonds’.^63^ At the same time, though, she holds that restoration or reparation is not a matter of ‘straightforward importation of civil into criminal law’.^64^ Rather, it is concerned with ‘a wider set of aims’ and ‘involves more than “making good” the damage done to property, body or psyche’.^65^ It ‘must also entail recognition of the harm done to the social relationship between offender and victim, and the damage done to the victim’s social rights in his or her property or person’.^66^

A restorative conception of rehabilitation can also be attributed to John Braithwaite and Philip Pettit, who hold that an aim of criminal justice is to restore ‘dominion’.^67^ They explain that

For dominion to be restored, what is sought is some evidence of a change in attitude, some expression of remorse that indicates that the victim’s rights will be respected in the future. Achieving such a change in attitude may entail the offender agreeing to undergo training, counselling or therapy and, as such, these may all be seen as part of reparative justice. A forced apology or obligatory payment of compensation will not suffice; indeed, it may even be counterproductive in eliciting a genuine change of attitude in the offender.^68^

## Means-Based Subvariants of the Conceptions

In the previous section, we distinguished five different conceptions of rehabilitation on the basis of their aims or ends. In relation to the first two conceptions—*rehabilitation as anti*-*recidivism* and *rehabilitation as harm*-*reduction*—we introduced a condition restricting the means that could be used to achieve the intended aim. In respect of the latter three conceptions—*rehabilitation as therapy, rehabilitation as moral improvement,* and *rehabilitation as restoration*—we included no such condition. This is because in respect of the first two conceptions, such a condition was needed to distinguish rehabilitation from incapacitation and deterrence. In respect of the latter, we do not think a means-based condition is necessary to distinguish rehabilitation from other functions of criminal justice. Nevertheless, some proponents of these latter three conceptions may also wish to impose means-based constraints on what can qualify as rehabilitation—or permissible rehabilitation—and we can distinguish subvariants of these views by reference to the nature and stringency of these constraints. For example, we can distinguish between views according to which an intervention must, if it is to qualify as (permissible) rehabilitation, engage the offender’s rational faculties (for example, by employing education programmes), views on which it may bypass the offender’s rational faculties but must engage other psychological processes (for example by employing forms of behavioural therapy that work largely at a subconscious level), and views on which rehabilitation may bypass psychological processes entirely, acting directly on neurochemical states (for example, through the administration of psychopharmaceuticals).

We have already hinted at such subvariants of *rehabilitation as moral improvement.* Several proponents of the moral improvement of criminal offenders endorse a requirement that attempts at moral improvement must employ means that engage the offender’s rationality. That is, they impose an ‘rationality constraint’ on the types of means that can permissibly be employed in rehabilitation; they reject as impermissible interventions that affect recipients in a way that bypasses their rationality (which we take to be ‘all mental processes that are rational, in the sense of being reasons-responsive’),^69^ for example because the ‘*initial* effects of the intervention on the motivational states of the recipient are not mediated by rational processes’.^70^

Morris and Hampton think that the moral improvement sought by institutions of criminal justice ought to come about as a result of autonomous action on the part of the offender and ought not to bypass ‘the human capacity for reflection, understanding, and revision of attitude’.^71^ The change in dispositions in the offender ought to be the result of his autonomous reflection. Hampton, Morris, and Duff all hold that attempts at moral improvement should seek to bring it about that the offender (among other things) becomes convinced that his actions were wrong, feels guilty about his actions, and resolves not to perform similar actions again. Such transformations could potentially be produced through, for example, brain washing or conditioning, but these authors reject the use of such means.

Hampton specifies that interventions that bring about the moral improvement of offenders are ‘not intended as a way of conditioning a human being to do what society wants her to do (in the way that an animal is conditioned by an electrified fence to stay within a pasture)’, but to teach ‘the wrongdoer that the action she did (or wants to do) is forbidden because it is morally wrong and should not be done for that reason’.^72^ On Hampton’s view, the State does not only want to change offenders to avoid them behaving in certain ways; it ‘also wants … to get the human wrongdoer to reflect on the moral reasons for that barrier’s existence, so that he will make the decision to reject the prohibited action for moral reasons, rather than for the self-interested reason of avoiding pain’.^73^

Though they reject the label themselves, Hampton, Morris and Duff can—as we have seen—be characterised as proponents of rehabilitation on *rehabilitation as moral improvement*. However, a more fine-grained characterisation of their views would understand them as proponents of a subvariant of this conception, according to which moral improvement must be sought through rationality-engaging, and not rationality-bypassing, means.

The distinction between rationality-engaging and rationality-bypassing interventions might be relevant to other conceptions of rehabilitation too. The requirement to engage rational faculties has advocates in recent literature on the use of ‘neurointerventions’—or interventions that act directly on the brain—for crime-prevention purposes, not all of whom are proponents of *rehabilitation as moral improvement*. For example, Elizabeth Shaw holds that ‘[e]fforts to reform the offender should be through rational dialogue’ because ‘subjecting a person to direct brain interventions would amount to treating her as if she were a puppet, an automaton or a robot—as something less than human … In other words, it would “objectify” her’.^74^ Shaw does not herself commit to any of the particular conceptions of rehabilitation that we delineate above. However, we could imagine that proponents of any of the conceptions that we have outlined might wish to invoke a reason-engagingness requirement that would render certain means incompatible with its conception of rehabilitation. Moving beyond the criminal justice literature, such a requirement has also been advocated in relation to, for example, the treatment of depression, where it is has figured in arguments for preferring psychotherapy to anti-depressants.^75^

There are further distinctions that can be made between subvariants of the different conceptions of rehabilitation based on the means they take to be consistent with (permissible) rehabilitation. For example, one of the present authors (Douglas) has distinguished between perceptual and non-perceptual influences, that is, interventions whose primary motivational effects are mediated by perceptual processes, and interventions where this is not the case.^76^ Some interventions bring about their motivational effects via perceptual mechanisms in the recipient, for example, the recipient seeing an aggression-attenuating colour or other environmental stimuli, which ‘then sets in train some brute subconscious process which attenuates strong impulses towards aggression’.^77^ In other interventions, there might be ‘no such role for perception’, because ‘motivational change is instead the upshot of a chemically or physically induced change in the neurochemical bases of aggression’.^78^ Douglas rejects the moral significance of the distinction, but notes that some might argue that, whereas perceptual means to rehabilitation can be permissible, non-perceptual means cannot, because they operate (bring about their intended motivational effects) in a way that is non-transparent to the recipient, or is difficult for the recipient to monitor, or is irresistible.^79^

Jan Christoph Bublitz and Reinherd Merkel distinguish between interventions that ‘bypass’ psychological processes altogether (which they call ‘direct’ interventions) and those whose effects are ‘mediated…by internal processes on the part of the addressee’ (which they call ‘indirect’ interventions).^80^ As they understand it, this distinction is not co-extensive with either the distinction between rationality-engaging and rationality-bypassing interventions, or that between perceptual and non-perceptual interventions. On the one hand, Bublitz and Merkel take perceptual mediation to be necessary, but perhaps not sufficient, for an intervention to qualify as indirect. They write that ‘[t]entatively, indirect (or external) interventions are those stimuli which are perceived sensually…and pass through the mind of the person, being processed by *a host of psychological mechanisms*’.^81^ On the other hand, they take rational mediation not to be necessary for indirectness, noting that the psychological processes engaged by indirect interventions, and bypassed by direct ones, are ‘not necessarily rational’.^82^ Bublitz and Merkel hold that both direct and indirect interventions are ‘stimuli changing mental states and, in whatever way they achieve this by, are always accompanied by changes in the brain’, but that indirect interventions are unable to bypass the recipient’s psychology and therefore respects him as a subject, whereas direct interventions that bypass the recipient’s psychology do not. Among permissible interventions, Bublitz and Merkel mention conscious or direct communication and psychotherapy, while they take impermissible direct interventions to include the administration of psychoactive substances and deep brain stimulation.^83^

As with the distinction between rationality-engaging and rationality-bypassing interventions, requirements to engage perception or to employ indirect means may potentially be used to generate further means-based subvariants of each of the conceptions of rehabilitation we identified in Sect. 3.

## Payoffs of Taxonomy

Delineating the five different ends-based conceptions of criminal rehabilitation identified by our taxonomy, and further means based subvariants, has, we think, at least two payoffs.

### Defining the Scope of Objections

One payoff is that the taxonomy helps to define the scope of some objections to rehabilitative theories of criminal justice. Delineating different conceptions of rehabilitation makes it clear which conceptions are, and are not, susceptible to common criticisms of rehabilitation. One influential criticism of the view that rehabilitation is a legitimate function of criminal justice, and an important reason that such views have fallen out of favour in moral and legal philosophy, is the ‘theoretical objection’ mentioned above—that rehabilitation fails to treat offenders as responsible moral agents.^84^ Our taxonomy suggests that this objection is more limited in its scope than proponents have seemed to assume.

There are two reasons why a rehabilitative intervention might fail to treat the offender as a rational agent: (i) because the intervention has an aim that is incompatible with viewing the offender as a full or adequate rational agent, or (ii) because the intervention employs means that fail to engage the offender’s rational agency, thereby failing to treat him as a full moral agent. If the objection is based on (i), it seems to apply primarily to *rehabilitation as therapy*, on which rehabilitation presupposes a mental deficit. Insofar as a mental deficit implies a lack of mental capacity, this view arguably presupposes that the recipient of the rehabilitation is less than fully responsible (though this will depend on which incapacities exactly are implied—the objection will have its fullest force in relation to what we called the forensic understanding of *rehabilitation as therapy*, since, on this understanding, rehabilitation targets precisely those mental capacities that are relevant to criminal responsibility). Other conceptions of rehabilitation are not vulnerable to this objection, since they do not presuppose any mental incapacity or lack of rational agency.

Perhaps *rehabilitation as moral improvement* and *rehabilitation as anti*-*recidivism* presuppose that the target of rehabilitation is flawed in some way.^85^ However, there is no reason to suppose that the flaw must be a lack of capacity rather than, say, a lack of moral virtue or the presence of immoral motives. Hampton’s view, for example, explicitly rejects the idea that offenders are individuals suffering from some illness or deficit for which they ought to receive treatment. She conceives of offenders as responsible moral agents who have acted immorally and to which punishment sends the moral message that they have acted immorally.^86^

Howard’s fortificationist view is presented as an attempt to overcome the class of objections according to which rehabilitation fails to treat offenders as responsible moral agents.^87^ He takes agents to be under a duty to fortify their moral capacities such that they do not commit criminal offences, and rehabilitation’s aim to be to foster those capacities. These capacities, in the criminal justice context, relate to what John Rawls describes as our first moral power: to ‘identify and be moved by moral duties of justice’, and have both an epistemic component, relating to ‘the identification of one’s justice-related moral duties’, and a motivational component, relating to ensuring ‘one’s compliance with those duties’.^88^ Offenders are, on Howard’s view, under an obligation to fortify their own moral capacities by undergoing rehabilitation as a matter of what they owe to their fellow moral agents. Far from being treated as not responsible for their criminal offences, offenders are on Howard’s view responsible for their failing moral capacities, or for failing do what it takes to bring about a state of affairs in which they do not culpably commit a criminal offence.

Our taxonomy thus clearly shows that we can reject rehabilitation, as characterised by *rehabilitation as therapy* or certain subvariants thereof, for the reasons proponents of the theoretical objection give—that it fails to treat offenders as morally responsible agents—but deny that these concerns or criticisms apply to other conceptions of rehabilitation and perhaps thereby maintain that rehabilitation (on these other conceptions) is a legitimate function of criminal justice.

If the objection is instead based on (ii)—that the intervention employs means that fail to engage the offender’s rational agency—then whether the objection succeeds depends on which means are used to pursue it. All of the conceptions of rehabilitation that we have introduced (including *rehabilitation as therapy*) are compatible with rehabilitation being pursued through rationality-engaging means, such as engaging an offender in rational dialogue. As noted above, existing views that see moral improvement as a legitimate function of criminal justice typically impose a ‘rationality constraint’ on the types of means that can permissibly be used for rehabilitation purposes, such that the means used must not bypass the offender’s rational capacities. But, as we noted, such a constraint could also be included in other conceptions, including *rehabilitation as therapy,* giving defenders of rehabilitation a way of avoiding objections based on (ii).

### Suggesting Connections to Other Literatures

A second payoff of our taxonomy is that it helps to draw links with other literatures by suggesting parallels between rehabilitation and other types of intervention. For example, on *rehabilitation as anti*-*recidivism* and *rehabilitation as harm*-*reduction*, rehabilitation is somewhat similar to some public health interventions, such as behaviour change campaigns intended to protect public health (for example, drink driving campaigns, vaccination promotion campaigns), suggesting that literature from public health might fruitfully inform discussions of rehabilitation. Parallels between criminal justice and public health have already received some attention, but these have focussed on quarantine,^89^ which, since it operates via the imposition of external constraints, is more analogous to incapacitation than rehabilitation. Other types of public health intervention, such as health promotion campaigns intended to encourage vaccination or social distancing, are more closely analogous to rehabilitation.

There are further possible links with other literatures that have not been explored, or which warrant further attention. On *rehabilitation as therapy*, for example, rehabilitation is in some respects similar to standard medical treatment, suggesting that literature from medical and psychiatric ethics—and especially on non-consensual psychiatric interventions—might be relevant to the discussion of rehabilitation. On *rehabilitation as moral improvement,* rehabilitation is relevantly similar to, for instance, the moral education of children, which standardly also aims at moral improvement. Again, this is a topic on which there is also some existing ethical discussion.^90^ Drawing these links may help to clarify the types of interventions that can permissibly be used to rehabilitate offenders, and constraints that ought to be placed on their use. For example, a consent requirement in relation to rehabilitative interventions is suggested by *rehabilitation as therapy*, given that consent is standardly required for medical therapies, but not by some other conceptions, such as *rehabilitation as anti*-*recidivism*.

Identifying connections to other literatures may also strengthen the case for rehabilitation. On any of our conceptions of rehabilitation there are, as we have suggested, practices analogous to rehabilitation outside the criminal justice context. These include, most obviously, health promotion interventions in public health, psychiatric treatments, and the moral education of children. This puts some pressure on opponents of rehabilitation to either (i) say something about why rehabilitation is inappropriate in criminal justice while these other interventions are appropriate outside the criminal justice context, or (ii) hold that these other interventions are inappropriate too. In the case of the comparison to the moral education of children, opponents of rehabilitation views could perhaps quite easily identify morally relevant differences; many accept that we can treat children in ways in which it would not be permissible to treat adults, for example, because children are yet to develop some capacities or agency or will that warrants the kind of respect we afford adults who are full moral agents, or because children have a different profile of prudential values.^91^ But identifying morally relevant differences is more difficult when the comparison is to practices that do not involve children.

Identifying links to other literatures may also help us make headway towards greater clarity in discussions of rehabilitation. To some extent, existing unclarity can be attributed to the fact that different conceptions of rehabilitation invoke notions, such as mental disorder and moral improvement, that are themselves open to multiple interpretations and frequently used imprecisely. This source of unclarity remains even when different conceptions of rehabilitation are distinguished. However, our taxonomy also suggests that we may be able to mitigate some of this unclarity by drawing on conceptual work done in other areas. For example, when defining mental deficit we might derive some benefit from work in psychiatry, the philosophy of mind, and the philosophy of science; when clarifying the moral improvement conception, we might rely on work on moral education and moral bioenhancement. Once we have achieved greater clarity in regard of what these notions mean or have better defined them, we can proceed to examine the extent to which they are measurable and how.

## Work to be Done

The concept of rehabilitation is often deployed in academic discussions, policy documents and legal judgments without being precisely defined, and without its extension being intuitively clear.

In this article, we have sought to bring a measure of clarity by offering a simple taxonomy of rehabilitation. We have outlined five conceptions of rehabilitation that can be discerned in the literature, as well as a number of subvariants of these conceptions. The five conceptions are distinguished from one another chiefly by the *ends* that they ascribe to rehabilitation, but subvariants within these conceptions are in some cases distinguished also by the *means* used to achieve these ends.

This taxonomy is, however, just a beginning. There may be scope to broaden our taxonomy by adding conceptions of rehabilitation that we have missed. And there is certainly scope to deepen it by distinguishing variants of the conceptions that it posits. One way to achieve such a deepening would be to more finely specify the aims of rehabilitative interventions, as we began to do with interventions that aim at moral improvement, for example, by distinguishing local and global forms of moral improvement. Similarly, different variants of *rehabilitation as anti*-*recidivism* could differ in the breadth or range of the types of criminal (and other) behaviour that they seek to prevent. Another approach would be to specify hierarchies of aims. For example, we could distinguish between views according to which rehabilitation aims at moral improvement *with the further aim of protecting the public* and views according to which moral improvement is seen as the ultimate end. Finally, yet another approach would be to introduce further means-based distinctions. Perhaps, for example, an interesting distinction could be drawn between effortful and effortless means.^92^ The broadening and deepening of our taxonomy is, however, a task for further work.

There are also difficult questions of application raised by our taxonomy: not all existing conceptions of rehabilitation can be neatly classified into the (overlapping) categories that it establishes. To give just one example, Plato describes a view that might be best understood as a mixture of *rehabilitation as therapy* and *rehabilitation as moral improvement*, on which an individual who has committed a wrong

should voluntarily go to wherever he will pay the penalty as soon as possible, to the judge as if to the doctor, eager to take care that the disease of wrongdoing not become chronic and make his soul fester and become incurable … He ought not to hide his injustice but bring it out in the open, so that he may pay his due and become well, and it is necessary for him not to act cowardly but to shut his eyes and be courageous, as if he were going to a doctor for surgery or cautery, pursuing the good and noble and taking no account of the pain, and if his injustice is worthy of a beating, he should put himself forward to be beaten.^93^

In Plato’s case it is hard to say whether the deficit to be corrected is a moral one (in which case his view might be treated as a variant of *rehabilitation as moral improvement*) or a prudential one (in which case it is perhaps closer to *rehabilitation as therapy*). This is unsurprising, given that ancient philosophers typically did not distinguish between prudence and morality.^94^ Plato’s view is an example of a view that does not fit neatly into our taxonomy, suggesting our taxonomy needs to be developed further. Again, we leave these questions as a possible subject for future work.

Our taxonomy leaves much work to be done, in further specifying the preliminary conceptions of rehabilitation that it offers, in teasing out the relationships between them, and perhaps in adding further conceptions. Nevertheless, we hope that it will serve as a useful starting point for further work on the nature of rehabilitation, and that it already makes some progress towards clarifying this ambiguous concept and the messy literature that surrounds it.

## Footnotes

1 See e.g. Andrew Ashworth, Andrew von Hirsch, Julian Roberts (eds.), *Principled Sentencing: Readings on Theory and Policy, 3rd ed* (Hart Publishing, 2009); Peter Raynor and Gwen Robinson, “Why help offenders? Arguments for rehabilitation as a penal strategy”, *European Journal of Probation* 1 (2009), pp. 3–20.

2 E.g. Ministry of Justice, *Transforming Rehabilitation. A Strategy for Reform,* May 2013, available at https://consult.justice.gov.uk/digital-communications/transforming-rehabilitation/results/transforming-rehabilitation-response.pdf; Ministry of Justice, *Prison Safety and Reform,* November 2016, available at https://assets.publishing.service.gov.uk/government/uploads/system/uploads/attachment_data/file/565012/cm-9350-prison-safety-and-reform-_print_.pdf. For some examples of legal judgments that emphasise the importance of rehabilitation, see note 11 below.

3 Ted Honderich, *Punishment. The Supposed Justifications Revisited* (London: Pluto Press 2006), p. 112.

4 Throughout, we understand rehabilitation as a type of intervention, rather than as a type of psychological process, though obviously the term ‘rehabilitation’ is used to refer to both.

5 James Rachels, “Punishment and Desert”, in Hugh LaFollette (ed) *Ethics in Practice* (Oxford: Blackwell, 1997), pp. 470–479.

6 Honderich, *Punishment. The Supposed Justifications Revisited*, p. 112; Steven Sverdlik, “Punishment and Reform”, *Criminal Law and Philosophy* 8 (2014): 619–633; Robert Nozick, *Philosophical Explanations* (Harvard University Press, 1981); Antony Duff, A. (2005) “Punishment and Rehabilitation—or rehabilitation as punishment”, *Criminal Justice Matters* 60 (2005): pp. 18–19; Victor Tadros, *The Ends of Harm. The Moral Foundations of Criminal Law* (Oxford: Oxford University Press, 2011).

7 For an argument to this effect, see Lucia Zedner, “Dangers of Dystopias in Penal Theory”, *Oxford Journal of Legal Studies* 2 (2002), pp. 341–366, at pp. 345–346. See also Edward L. Rubin, “The Inevitability of Rehabilitation”, *Law & Inequality: A Journal of Theory and Practice* 19 (2001), pp. 343–377.

8 E.g. Aggression Replacement Training, a programme designed for individuals ‘convicted of violent offences or who have problems controlling their temper’. The programme ‘challenges offenders to accept responsibility for their behaviour; the aims are to reduce the incidence of assault, public order offences and criminal damage, increase public protection and challenge offenders to accept responsibility for their crime and its consequences’. Another similar programme is Controlling Anger and Learning to Manage it (CALM), which is an ‘emotional management programme designed for those whose offending behaviour is precipitated by intense emotions’, the goal of which is to ‘assist offenders understand the factors that trigger their anger and aggression and learn skills to manage their emotions’. See Ministry of Justice, ‘Offender Behaviour Programmes (OBPs)’ https://www.justice.gov.uk/offenders/before-after-release/obp accessed 30 December 2017.

9 E.g. FOCUS Substance misuse programme and Addressing Substance Related Offending (ASRO), both of which are cognitive behavioural intervention programmes aimed at addressing individuals’ alcohol or drug related offending behaviour, see Ministry of Justice, ‘Offender Behaviour Programmes (OBPs)’ https://www.justice.gov.uk/offenders/before-after-release/obp accessed 30 December 2017.

10 An example of the latter is the Sex Offenders Treatment Programme—Core (SOTP Core), which ‘helps offenders develop understanding of how and why they have committed sexual offences [and] increases awareness of victim harm’. SOTP Core’s ‘main focus is to help the offender develop meaningful life goals and practice new thinking and behavioural skills that will lead him away from offending’, see Ministry of Justice, ‘Offender Behaviour Programmes (OBPs)’ https://www.justice.gov.uk/offenders/before-after-release/obp accessed 30 December 2017.

11 Sonja Meijer, “Rehabilitation as a Positive Obligation”, *European Journal of Crime, Criminal Law and Criminal Justice* 25 (2017): 145–162. See e.g. the cases of *Murray v Netherlands (Application 10511/10)* (2017) 64 E.H.R.R. 3, para 104 and *Khoroshenko v. Russia (Application no. 41418/04)*, 30 June 2015, para. 121. The importance of rehabilitation is also emphasised in European Court of Human Rights jurisprudence such as *Dickson v United Kingdom (Application No.44362/04)* (2008) 46 E.H.R.R. 41, para. 75*; Vinter and others v. United Kingdom (Application no. 66069/09)* (2016) 63 E.H.R.R. 1, para. 115 and *Harakchiev and Tolumov v. Bulgaria*, 8 July 2014, paras. 243–246.

12 Jeffrey Howard, “Punishment as Moral Fortification”, *Law and Philosophy* 36 (2017): 45–75.

13 Howard, “Punishment as Moral Fortification”.

14 Howard, “Punishment as Moral Fortification”.

15 Howard, “Punishment as Moral Fortification”, p. 59. See also Doris Layton MacKenzie, *What Works in Corrections: Reducing the Criminal Activities of Offenders and Delinquents* (Cambridge: Cambridge University Press, 2006); Francis T. Cullen and Karen E. Gilbert, *Reaffirming Rehabilitation, 2nd ed* (Routledge 2013): 201–208.

16 Richard Moran, “Medicine and crime: The search for the born criminal and the medical control of criminality”, in Peter Conrad and Joseph W. Schneider, *Deviance and Medicalization* (Temple University Press, 1992) pp. 215–240, at p. 223; Thomas Douglas, “Criminal Rehabilitation Through Medical Intervention: Moral Liability and the Right to Bodily Integrity”, *Journal of Ethics* 18 (2014): 101–122, at pp. 101-102. These types of interventions have become the subject of moral debate, see e.g. Elizabeth Shaw, “Direct Brain Interventions and Responsibility Enhancement”, *Criminal Law and Philosophy* 8 (2014): 1-20; Douglas T, “Nonconsensual Neurocorrectives and Bodily Integrity: A Reply to Shaw and Barn”, *Neuroethics* 12 (2019): 107–118.

17 Antony Duff, *Punishment, Communication and Community* (New York: Oxford University Press, 2001), p. 5; Zachary Hoskins, “Punishment, Contempt, and the Prospect of Moral Reform”, *Criminal Justice Ethics* 32: 1-18, at p. 9.

18 Fergus McNeill, “Punishment as rehabilitation”, in Gerben Bruinsma and David Weisburd (eds.) *Encyclopedia of Criminology and Criminal Justice* (New York: Springer, 2014), pp. 4195-4206; Peter Raynor and Gwen Robinson, *Rehabilitation, Crime and Justice* (Palgrave Macmillan 2009).

19 Sverdlik, “Punishment and Reform”, p. 620.

20 Sverdlik, “Punishment and Reform”; J.M.E. McTaggart, *Punishment. Studies in Hegelian Cosmology* (2nd ed) (Cambridge: Cambridge University Press, 1918): 129–50, 132f.

21 Sverdlik, “Punishment and Reform”. See Jeremy Bentham, *An Introduction to the Principles of Morals and Legislation* (London: Methuen, 1982), p. 180–1; A.C. Ewing, *The Morality of Punishment* (London: Kegan, Paul, Trench, Trubner, 1929).

22 Sverdlik, “Punishment and Reform”.

23 Herbert Morris, “A Paternalistic Theory of Punishment”, *American Philosophical Quarterly* 18 (1981): 263–71, at p. 264.

24 Jean Hampton, “The Moral Education Theory of Punishment”, *Philosophy and Public Affairs* 13 (1984): 208–38, at pp. 214-215.

25 Duff, *Punishment, Communication and Community*, pp. 90–1.

26 Lene Bomann-Larsen, “Voluntary Rehabilitation? On Neurotechnological Behavioural Treatment, Valid Consent and (In)appropriate Offers”, *Neuroethics* 6 (2013): 65–77, at p. 65.

27 Shaw, “Direct Brain Interventions and Responsibility Enhancement”.

28 Douglas, “Criminal Rehabilitation Through Medical Intervention”.

29 E.g. Shaw, “Direct Brain Interventions and Responsibility Enhancement”; Kasper Lippert-Rasmussen, “The Self-Ownership Trilemma, Extended Minds, and Neurointerventions”, in David Birks and Thomas Douglas, *Treatment for Crime* (Oxford: Oxford University Press, 2018), pp. 140–158.

30 McNeill, “Punishment as rehabilitation”.

31 Raynor and Robinson, *Rehabilitation, Crime and Justice* 2009, p. 4.

32 Meijer, “Rehabilitation as a Positive Obligation”, p. 146.

33 Not all agree that rehabilitation or reform should be distinguished from special deterrence. See, for example, Arnold S. Kaufman, “The Reform Theory of Punishment”, *Ethics* 71 (1960): 49-53, at p. 49.

34 E.g. Ministry of Justice, *Transforming Rehabilitation. A Strategy for Reform,* May 2013, available at https://consult.justice.gov.uk/digital-communications/transforming-rehabilitation/results/transforming-rehabilitation-response.pdf. Phelps argues there has been a rhetorical shift in the US so that rehabilitation now refers to anti-recidivism: Michelle Phelps, “Rehabilitation in the Punitive Era: The Gap between Rhetoric and Reality in U.S. Prison Programs”, *Law & Society Review* 45 (2011), pp. 33–68.

35 Sverdlik, “Punishment and Reform”, p. 628.

36 Sverdlik’s view is like some of the moral improvement views that we will consider later on in that it sees rehabilitation as something that should be aimed at improving offenders’ reasons-responsiveness, but it is unlike these moral improvement views in that it does not take the reasons rehabilitation aims to improve to be just moral reasons; efforts at improving reasons-responsiveness on his view can also aim at prudential reasons. Sverdlik thinks that the requirement that offenders should refrain from offending for moral reasons is overly demanding, and that it is imprudent for those who seek to defend rehabilitation as an aim of criminal justice to insist on moral motivation in offenders, since it is (1) hard to measure, (2) does not necessarily lead to reduced recidivism, (3) overly demanding since it might exclude some instances of successfully induced anti-recidivism where offenders obey the law for self-interested reasons. (But he allows for insistence on moral motivation insofar as acting from moral motivation makes offenders more stably disposed to acting in ways that does not affect others’ well-being negatively.)

37 Bertrand Russell, *Roads to Freedom* (London: George Allen and Unwin Ltd, 1918) at p. 135. For another defence of *rehabilitation as therapy,* see Karl Menninger, *The Crime of Punishment* (Viking. 1969).

38 Hampton, “The Moral Education Theory of Punishment”, pp. 214–215.

39 Morris, “A Paternalistic Theory of Punishment”, p. 266. He also discusses the reasoning justifying *rehabilitation as therapy* in Herbert Morris, “Persons and Punishment”, *The Monist* 52 (1968): 475-501, at pp. 480–488.

40 Sverdlik, “Punishment and Reform”, p. 261.

41 *Rehabilitation as therapy* characterises the goals of rehabilitation normatively *if* the concepts of mental illness and mental deficit are themselves normative.

42 Hampton, “The Moral Education Theory of Punishment”, p. 213.

43 Hampton seems to take what she refers to as rehabilitation theories to be something like our *rehabilitation as anti-recidivism* or *rehabilitation as cost-reduction* conceptions. In her view, rehabilitation theories take the good to be ‘the wrongdoer’s acceptance of society’s mores and her successful operation in the community’: Hampton, “The Moral Education Theory of Punishment”, p. 215.

44 Sverdlik, “Punishment and Reform”, p. 261.

45 Duff, *Punishment, Communication and Community*, p. 19.

46 Howard, “Punishment as Moral Fortification”, p. 61.

47 Howard, “Punishment as Moral Fortification”.

48 Thomas Douglas, “The Morality of Moral Neuroenhancement”, in Jens Clausen and Neil Levy (eds.) *Handbook of Neuroethics* (Springer, 2015).

49 Sverdlik, “Punishment and Reform”. Sverdlik argues that this also applies to Duff.

50 Sverdlik, “Punishment and Reform”, p. 623.

51 But note that the scope of the moral improvement that could permissibly be aimed at is restricted to individuals who have previously committed a criminal offence, see Russ Shafer-Landau, “Can Punishment Morally Educate?”, *Law and Philosophy* 10 (1991), pp. 191–192.

52 Morris, “A Paternalistic Theory of Punishment”, p. 265.

53 Morris, “A Paternalistic Theory of Punishment”, p. 265.

54 Howard, “Punishment as Moral Fortification”.

55 Hampton, “The Moral Education Theory of Punishment”, pp. 213–214.

56 Hampton, “The Moral Education Theory of Punishment”, p. 216. For a discussion regarding the scope of moral education and the range of behaviour that the state may legitimately punish, see Hampton, pp. 218–220.

57 Sverdlik, “Punishment and Reform”, p. 621.

58 Sverdlik, “Punishment and Reform”, p. 625.

59 Duff, *Punishment, Communication and Community*, p. 126. Sverdlik takes this to mean that Duff would ‘allow for efforts to transform an offender’s general attitudes towards, say, property rights, even if he was only convicted of burglary. But it would seem to disallow efforts at transforming this offender’s attitudes towards spousal abuse if he was only convicted of burglary.’ Sverdlik, “Punishment and Reform”, p. 625.

60 The terms reparation and restoration are also used with different meanings in different contexts and by different authors and, as Lucia Zedner notes, ‘it is far from clear that they share a common vision as to its shape and purpose’: Lucia Zedner, “Reparation and Retribution: Are They Reconcilable?” *Modern Law Review* (1994): 228-250, at p. 234. See also Kathleen Daly and Gitana Proietti-Scifoni, “Reparation and Restoration”, in Michael Tonry (ed.), *The Oxford Handbook of Crime and Criminal Justice* (Oxford University Press, 2013) pp. 207–253.

61 Zedner, ‘Reparation and Retribution: Are They Reconcilable?’, pp. 235–238. See also John Braithwaite, *Crime, Shame and Reintegration* (Cambridge University Press, 1989).

62 Margaret Fry, *Arms of the Law* (Victor Gollancz, 1951), p. 126).

63 Zedner, ‘Reparation and Retribution: Are They Reconcilable?’, p. 233.

64 Zedner, ‘Reparation and Retribution: Are They Reconcilable?’, p. 234.

65 Zedner, ‘Reparation and Retribution: Are They Reconcilable?’, p. 234.

66 Zedner, ‘Reparation and Retribution: Are They Reconcilable?’, p. 234.

67 John Braithwaite and Philip Pettit, *Not Just Deserts: A Republican Theory of Criminal Justice* (Oxford University Press, 1990), p. 37.

68 Zedner, ‘Reparation and Retribution: Are They Reconcilable?’, pp. 234–235.

69 Thomas Douglas, “Neural and Environmental Modulation of Motivation. What’s the Moral Difference?”, in David Birks and Thomas Douglas (eds.) *Treatment for Crime: Philosophical Essays on Neurointerventions in Criminal Justice* (Oxford University Press, 2018): 208-223, at p. 215. For a discussion of this objection see Douglas, pp. 215–217.

70 Douglas, “Neural and Environmental Modulation of Motivation. What’s the Moral Difference?”, p. 216. See also Thomas Douglas, “Enhancing Moral Conformity and Enhancing Moral Worth”, *Neuroethics* 7 (2014): 75–91.

71 Morris, “A Paternalistic Theory of Punishment”, p. 265; Hampton, “The Moral Education Theory of Punishment”, p. 222; Duff, *Punishment, Communication and Community*, p. 122.

72 Hampton, “The Moral Education Theory of Punishment”, p. 212.

73 Hampton, “The Moral Education Theory of Punishment”, p. 212.

74 Elizabeth Shaw, “Direct Brain Interventions and Responsibility Enhancement”, *Criminal Law and Philosophy* 8 (2014): 1–20. Robert Sparrow makes the same argument in Robert Sparrow “Better Living through Chemistry?”, *Journal of Applied Philosophy* 31 (2014): 23–32, at pp. 26–27.

75 See e.g. Carl Elliott, “The tyranny of happiness: Ethics and cosmetic psychopharmacology”, in Erik Parens (ed.) *Enhancing human traits: Ethical and social implications* (Georgetown University Press, 1998): 177–188. For a discussion see Neil Levy, “Rethinking Neuroethics in the Light of the Extended Mind Thesis”, *American Journal of Bioethics* 7 (2007): 3-11, at pp. 7-10 and Neil Levy, *Neuroethics: Philosophical challenges for the 21st century* (Cambridge University Press, 2007).

76 Douglas, “Neural and Environmental Modulation of Motivation. What’s the Moral Difference?”, p. 218. Note that Douglas discusses but does not endorse this distinction. There is room for significant debate over how to draw the distinction between rationality-engaging and rationality-bypassing interventions, see e.g. Neil Levy, “Nudge, Nudge, Wink, Wink: Nudging is Giving Reasons”, *Ergo* 6 (2019): 281-302, and also Neil Levy, “Nudges in a post-truth world”, *Journal of Medical Ethics* 43 (2017): 495–500 and Neil Levy, “Nudges to reason: not guilty”, *Journal of Medical Ethics* 44 (2018): 723.

77 Douglas, “Neural and Environmental Modulation of Motivation. What’s the Moral Difference?”, p. 218.

78 Douglas, “Neural and Environmental Modulation of Motivation. What’s the Moral Difference?”, p. 218.

79 Douglas, “Neural and Environmental Modulation of Motivation. What’s the Moral Difference?”, pp. 219–222.

80 Jan Christoph Bublitz and Reinhard Merkel, “Crimes Against Minds: On Mental Manipulations, Harms and a Human Right to Mental Self-Determination”, *Criminal Law and Philosophy* 8 (2014): 51–77, at pp. 69–70. Note that Neil Levy has in earlier work used the direct–indirect distinction to denote interventions that affect the recipient’s brains via her rational capacities (indirect) or bypassing them (direct): Neil Levy, *Neuroethics: Challenges for the 21*^*st*^ *Century* (Cambridge University Press, 2007), p. 70.

81 Bublitz and Merkel, “Crimes Against Minds”, pp. 69-70, our italics. See also Douglas, “Neural and Environmental Modulation of Motivation. What’s the Moral Difference?”, note 20.

82 Bublitz and Merkel, “Crimes Against Minds”, p. 70.

83 It might turn out that some of the distinctions relied on to generate means-based subvariants of the conceptions are untenable. Theorists have noted that a distinction between, for example, rationality-engaging and rationality-bypassing means is hard to sustain and that it is questionable whether, even if it could be sustained, it would track something of moral significance. See e.g. Douglas, “Neural and Environmental Modulation of Motivation. What’s the Moral Difference?”; Henry T. Greely, ‘Neuroscience and Criminal Justice: Not Responsibility but Treatment’, *Kansas Law Review* 56 (2008): 1103-38, at pp. 1133-34; Matt Matravers, “The Importance of Context in Thinking About Crime-Preventing Neurointerventions”, in David Birks and Thomas Douglas (eds.) *Treatment for Crime: Philosophical Essays on Neurointerventions in Criminal Justice* (Oxford University Press, 2018): 71-93, at pp. 82-83; Levy, “Nudge, Nudge, Wink, Wink: Nudging is Giving Reasons”.

84 Howard, “Punishment as Moral Fortification”.

85 The same may be true of some forms of *rehabilitation as harm-reduction*, insofar as they view the offender as needing to improve his prudential reasoning, or something like that.

86 Hampton, “The Moral Education Theory of Punishment”, pp. 214-215.

87 Howard, “Punishment as Moral Fortification”, p. 61.

88 Howard, “Punishment as Moral Fortification”, p. 49.

89 E.g. Derk Pereboom, *Living Without Free Will* (Cambridge: Cambridge University Press, 2001); Derk Pereboom, *Free Will, Agency, and Meaning in Life* (Oxford: Oxford University Press, 2014); Derk Pereboom, “A Defense of Free Will Skepticism: Replies to Commentaries by Victor Tadros, Saul Smilansky, Michael McKenna, and Alfred R. Mele on *Free Will, Agency, and Meaning in Life*”, *Criminal Law and Philosophy* 11 (2017): 617–636; Gregg D. Caruso, “Free Will Skepticism and Criminal Behavior: A Public Health-Quarantine Model,” *Southwest Philosophy Review* 32 (2016): 25-48; Gregg Caruso, “The Public Health-Quarantine Model”, in Dana Nelkin and Derk Pereboom (eds.), *Oxford Handbook of Moral Responsibility* (New York: Oxford University Press).

90 For a recent extended discussion, see Michael Hand, *A Theory of Moral Education* (Routledge, 2017).

91 E.g. Tamar Schapiro, “What Is a Child?”, *Ethics* 109 (1999): 715–738; Anthony Skelton, “Children's Well-being: A Philosophical Analysis”, in Guy Fletcher (ed.), *The Routledge Handbook of Philosophy of Well-being* (Routledge, 2015), pp. 366-377.

92 This distinction has sometimes been thought to have moral significance in discussions of biomedical enhancement, including moral bioenhancement, see e.g. Lisa Forsberg and Anthony Skelton, “Achievement and Enhancement”, *Canadian Journal of Philosophy* 50 (2020): 322–338; Thomas Douglas, “Enhancement and desert”, *Politics, Philosophy & Economics* 18 (2019): 3–22; Thomas Douglas, “Enhancing Moral Conformity and Enhancing Moral Worth”, Neuroethics 7 (2014): 75–91.

93 Plato, *Gorgias,* translated by Terence Irwin (Oxford University Press, 1979), p. 53.

94 Henry Sidgwick, The Methods of Ethics, 7^th^ ed (Macmillan, 1907), pp. 91-92. For a different view, see Terence Irwin, *The Development of Ethics: A Historical and Critical Study, Volume I: From Socrates to the Reformation* (Oxford University Press, 2007).

